# *Schistosoma mansoni* infection is associated with quantitative and qualitative modifications of the mammalian intestinal microbiota

**DOI:** 10.1038/s41598-018-30412-x

**Published:** 2018-08-13

**Authors:** Timothy P. Jenkins, Laura E. Peachey, Nadim J. Ajami, Andrew S. MacDonald, Michael H. Hsieh, Paul J. Brindley, Cinzia Cantacessi, Gabriel Rinaldi

**Affiliations:** 1 Department of Veterinary Medicine, University of Cambridge, Cambridge, CB3 0ES UK; 2 Alkek Center for Metagenomics and Microbiome Research, Department of Molecular Virology and Microbiology, Baylor College of Medicine, Houston, Texas USA; 3 Manchester Collaborative Centre for Inflammation Research, Division of Infection, Immunity and Respiratory Medicine, School of Biological Sciences, Faculty of Biology, Medicine and Health, University of Manchester, Manchester Academic Health Science Centre, Manchester, M13 9PL UK; 4 Biomedical Research Institute, Rockville, Maryland USA; 5 Department of Urology, School of Medicine and Health Sciences, George Washington University, Washington, USA; 6 Children’s National Health System, Washington, District of Columbia USA; 7 Department of Microbiology, Immunology & Tropical Medicine, and Research Center for Neglected Diseases of Poverty, School of Medicine & Health Sciences, George Washington University, Washington, DC 20037 USA; 8 Wellcome Sanger Institute, Wellcome Genome Campus, Hinxton, CB10 1SA UK

## Abstract

In spite of the extensive contribution of intestinal pathology to the pathophysiology of schistosomiasis, little is known of the impact of schistosome infection on the composition of the gut microbiota of its mammalian host. Here, we characterised the fluctuations in the composition of the gut microbial flora of the small and large intestine, as well as the changes in abundance of individual microbial species, of mice experimentally infected with *Schistosoma mansoni* with the goal of identifying microbial taxa with potential roles in the pathophysiology of infection and disease. Bioinformatic analyses of bacterial 16S rRNA gene data revealed an overall reduction in gut microbial alpha diversity, alongside a significant increase in microbial beta diversity characterised by expanded populations of *Akkermansia muciniphila* (phylum Verrucomicrobia) and lactobacilli, in the gut microbiota of *S*. *mansoni*-infected mice when compared to uninfected control animals. These data support a role of the mammalian gut microbiota in the pathogenesis of hepato-intestinal schistosomiasis and serves as a foundation for the design of mechanistic studies to unravel the complex relationships amongst parasitic helminths, gut microbiota, pathophysiology of infection and host immunity.

## Introduction

Schistosomiasis, a major neglected tropical disease, is considered the most problematic of the human helminthiases in terms of morbidity and mortality^1^. The causative agents are the blood flukes, trematodes of the genus *Schistosoma* including *S*. *mansoni*, *S*. *japonicum* and *S*. *haematobium*. Schistosomiasis in its several forms has been estimated to cause ≥3.5 million Disease Adjusted Life Years (DALYs)^2,3^. In Sub-Saharan Africa, 393 million people are estimated to be exposed to the parasite and, of these, 54 million suffer from overt schistosomiasis^4^. Humans are the definitive hosts of *S*. *mansoni*, and harbour the adult males and females, that live in the portal system and mesenteric veins^5,6^. The females shed eggs that traverse the lining of the mesenteric veins, proceed through the wall of the intestines, preferentially *via* the lymphoid tissue of the Peyer’s patches^7–9^ to the intestinal lumen and exit with the faecal stream. The eggs reach the fresh water environment and hatch a ciliated miracidium that seeks out and infects a suitable species of gastropod snail, e.g. species of the genus *Biomphalaria*. Within the snail, the parasites multiply *via* asexual reproduction, after which the cercarial developmental stage exits the snail. The fork-tailed cercaria is the infective stage for humans, who contract the infection in water contaminated with cercariae. During the skin penetration, the cercariae shed the tail and are henceforth known as schistosomula; these enter the circulation and are transported *via* the heart to the lungs and liver where they mature over several weeks. After about four weeks, the adult worms, which exhibit sexual dimorphism, migrate into the mesenteries of the intestines (*S*. *japonicum* and *S*. *mansoni*) or the blood vessels of the urinary bladder and other pelvic organs (*S*. *haematobium*), where they commence sexual reproduction releasing hundreds to thousands of eggs per day, depending on the species. These parasites can live for decades^3,6^.

The pathobiology of schistosomiasis is mostly associated with tissue lesions caused by migrating parasite eggs^10^. A large proportion of schistosome eggs fail to reach the intestinal lumen, instead becoming trapped in hepatic sinusoids and the intestinal wall, where they provoke formation of collagen-rich granulomas accompanied by periportal fibrosis and portal hypertension. Granuloma formation is mediated by host immunity to egg antigens; in particular, while a strongly polarised Th2-mediated immune response is responsible for the development of large granulomas during the initial phases of parasite establishment, chronic infections are accompanied by the onset of regulatory responses that lead to the formation of smaller granulomas around newly deposited eggs^7^. In the mouse model of infection with *S*. *mansoni*, the immuno-regulatory environment confers protection against hepatotoxicity (reviewed by^7^). Nevertheless, the immune-molecular mechanisms underlying these regulatory responses have yet to be fully defined. In particular, whereas *Schistosoma* egg antigens interact directly with splenic B cells in the mouse, leading to production of IL-10 and expansion of regulatory T cell (Treg) populations^11^, the contribution of environmental stimuli to the initiation of these immune-regulatory mechanisms is not well understood. Notably, in a mouse model of infection by the hookworm-like nematode *Nippostrongylus brasiliensis*, parasite-induced Th2 type immune responses are accompanied by profound alterations of the gut microbiota composition, including marked contraction of populations of segmented filamentous bacteria (SFB, Gram-positive members of the family *Clostridiaceae*) and down-regulation of pro-inflammatory IL-17^12^. In turn, infection of SFB-deficient mice results in unaltered IL-17 gene expression^12^, thereby supporting a key role for selected taxa of bacteria in helminth-driven modulation of immunity. Based on these observations, it seems plausible to hypothesise that the shift between Th2-type and regulatory responses that accompany egg production and characterises chronic schistosomiasis may be triggered, at least in part and directly or indirectly, by parasite-associated modifications in the composition of the intestinal commensal microbiota.

In spite of the burgeoning interest in understanding the complex interactions occurring at the host-parasite-microbiota interface^12–25^, studies investigating the impact of acute or chronic *S*. *mansoni* infection on the intestinal microbiota of its mammalian host are, thus far, scant. In a single study, Holzscheiter *et al*.^26^ demonstrated that administration of broad spectrum antibiotics and antimycotics to *S*. *mansoni*-infected C57BL/6 mice resulted in a substantial reduction of intestinal inflammation and intestinal granuloma development, thus providing support to a direct role of the host gut microbes in the pathogenesis of schistosomiasis. Here, we have directly addressed this issue by defining qualitative and quantitative fluctuations in intestinal microbial community profiles during infection with *S*. *mansoni*, and identified groups of bacteria with known roles in immune-modulation (e.g. lactobacilli), maintenance of epithelial barrier function integrity (i.e. *Akkermansia muciniphila*), and intestinal inflammation (e.g. *Dorea* and *Bacteroides acidifaciens*) that may play significant roles in the pathophysiology of acute and chronic schistosomiasis.

## Results

### Comparison of the gut microbiota composition in *Schistosoma mansoni*-infected mice *versus* uninfected controls at two time-points post-infection

**Figure 1:**
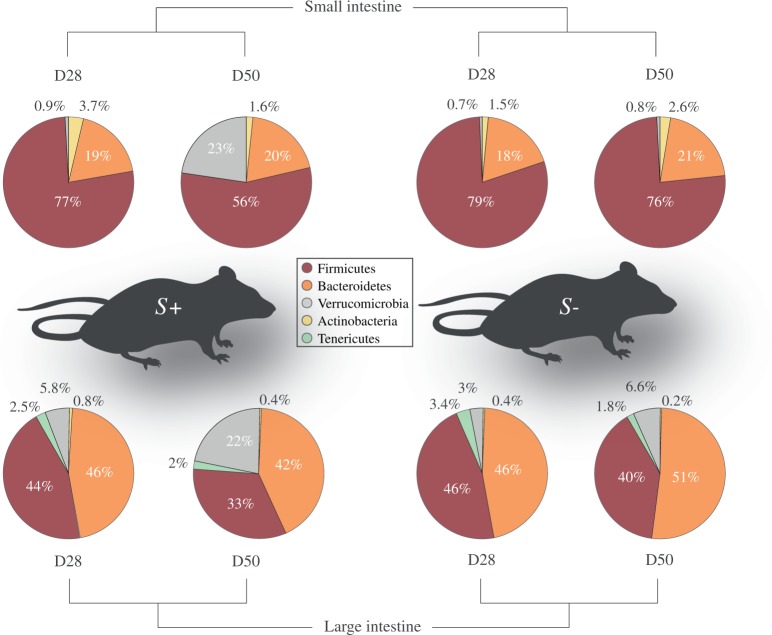
Bacteroidetes and Firmicutes are most abundant in the mouse gut microbiome. Relative abundances of bacterial phyla detected in luminal content samples from the small and large intestine of mice infected by *Schistosoma mansoni* (*S*+) at 28 and 50 days post-infection (D28 p.i. and D50 p.i., respectively), as well as of uninfected controls (*S−*). Percentages in individual pie chart sections indicate the relative proportion of the corresponding phylum.

Ten Swiss-Webster female mice (*S*+) were infected with 200 *S*. *mansoni* cercariae, while 10 age- and gender-matched mice remained uninfected and were included as controls (*S−*). For each *S*+ and *S−* group, five mice were euthanised 28 days after infection (D28 p.i., i.e. before egg laying by sexually mature *S*. *mansoni* females had commenced). The remaining mice (n = 5 for each *S*+ and *S−*) were euthanised at day 50 p.i. (D50 p.i., corresponding with ongoing egg laying by sexually mature *S*. *mansoni* females). An average of 40 mixed-sex adult parasites were recovered from individual *S*+ mice at D28 p.i., with comparable numbers being collected at D50 p.i. Luminal content samples were collected from sections of the small (SI) and large intestines (LI) of individual *S*+ and *S−* mice at each D28 p.i. and D50 p.i. From all samples analysed in this study (n = 80), a total of 1,305,817 paired-end reads were generated and subjected to further processing (Mendeley 10.17632/y8c7vpc8zp.1). No amplification was obtained from two no-template negative control samples (see Materials and Methods). Following filtering of singleton Operational Taxonomic Units (OTUs), duplicate sequences and chimeric sequences, a total of 974,491 high-quality reads (per sample mean: 12,181 ± 3,589) were retained. Rarefaction curves generated indicated that the majority of the bacterial communities were represented in the remaining sequence data (cf. Supplementary Fig. S1). These sequences were assigned to 8,734 OTUs and seven bacterial phyla (Mendeley DOI: doi:10.17632/y8c7vpc8zp.1). Overall, the phyla Firmicutes and Bacteroidetes were predominant in all samples analysed (54.2% average ± 23.9% standard deviation, and 34.9% ± 19.4%, respectively), followed by the phylum Verrucomicrobia (8% ± 13.4%) (Fig. 1). However, differences were observed in the relative abundance of Firmicutes and Bacteroidetes between samples from the SI and LI; in particular, while Firmicutes were significantly more abundant than Bacteroidetes in the SI throughout the course of the infection (73.1% ± 18.7% *vs* 19.5% ± 11.6 for Firmicutes and Bacteroidetes, respectively), similar proportions of the two phyla were detected in the LI (Firmicutes: 40.9% ± 16.6%; Bacteroidetes: 47.1% ± 15.5%) (Fig. 1). Verrucomicrobia were marginally less abundant in the microbiota from SI (5.1% ± 14.4%) than in that from LI samples (8.7% ± 12%) (Fig. 1).

### Gut microbial alpha diversity was reduced in the presence of *S*. *mansoni* infection at day 50 post-infection, whereas beta diversity increased over time

**Figure 2:**
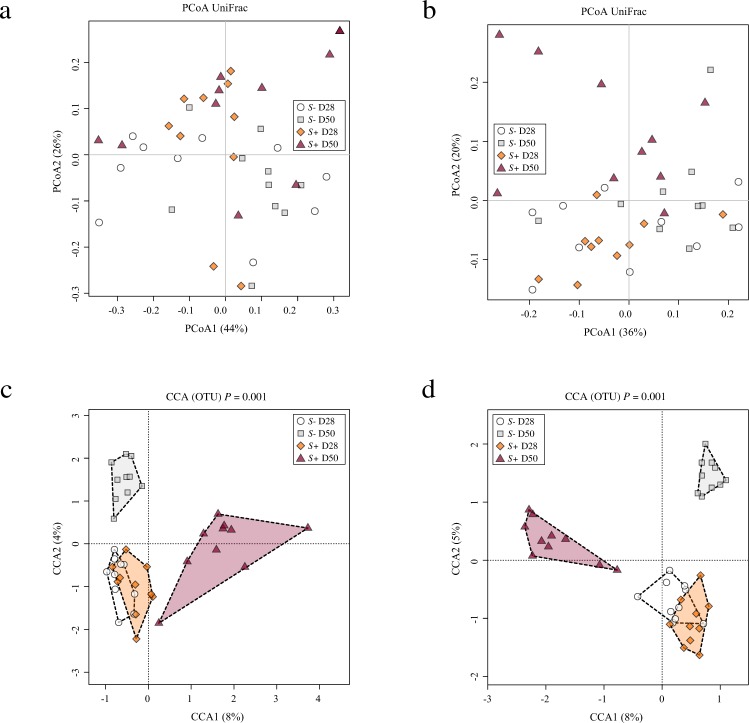
Profound shifts in the gut microbial profiles of *Schistosoma* *mansoni* infected mice were observed at 50 days post-infection. The gut microbial profiles of luminal content samples from the small and large intestine of mice infected by *S. mansoni* (*S*+) at 28 and 50 days post-infection (D28 p.i. and D50 p.i., respectively), as well as of uninfected controls (*S*−), ordinated by Principal Coordinates Analysis (PCoA) (**a**: small intestine; **b**: large intestine) and Canonical Correspondence Analysis (CCA) (**c**: small intestine; **d**: large intestine).

**Figure 3:**
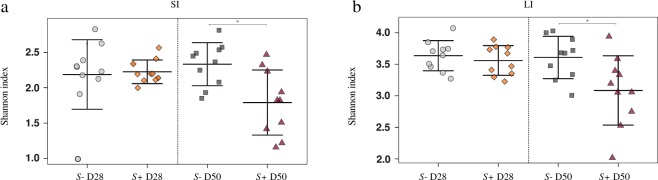
Gut microbial shannon diversity was significantly reduced in *Schistosoma* *mansoni* infected mice at 50 days post-infection compared to uninfected controls. Differences between microbial Shannon diversity detected in the gut microbiota of mice infected with *S. mansoni* (*S*+) at day 28 and 50 post-infection (D28 p.i. and D50 p.i., respectively) and that of uninfected control mice (*S*−), in the small (**a**; SI) and large intestine (**b**; LI). Asterisks denote significant differences (*P* < 0.05) between individual groups.

**Figure 4:**
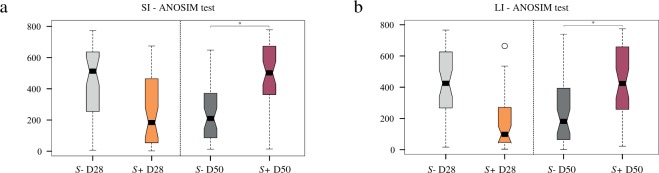
Gut microbial beta diversity was significantly increased in *Schistosoma mansoni* infected mice 50 days post-infection compared to uninfected controls. Differences between microbial beta diversity detected in the gut microbiota of mice infected with *S. mansoni* (*S*+) at day 28 and 50 post-infection (D28 p.i. and D50 p.i., respectively) compared with control mice (*S*−), in the small (**a**; SI) and large intestine (**b**; LI). Asterisks denote significant differences (*P* < 0.05) between individual groups.

Mouse gut microbial communities were clustered by Principal Coordinates Analysis (PCoA), which clearly separated samples from *S*+ mice at D50 p.i. from all other samples (Fig. 2). Although differences between SI and LI microbial community profiles of *S*+ and *S−* mice at each time-point were statistically significant when analysed through Canonical Correspondence analyses (CCA) (SI = *P* = 0.001; LI = *P* = 0.001), the largest difference was detected between the gut microbial compositions of SI and LI of *S*+ mice at D50 p.i. and the remaining sampling groups (Fig. 2). The portion of variability accounted for by explanatory variables in the CCA was 0.187 and 0.218 for the SI and LI, respectively (Supplementary Fig. S2). Analyses of Variance (ANOVA) of shannon diversity values obtained from the SI and LI of *S*+ and *S−* mice at D28 p.i. and D50 p.i. revealed a significant decrease in microbial alpha diversity at D50 p.i. in both intestinal sites in *S*+ mice (SI = *P* = 0.006; LI = *P* = 0.02; Fig. 3). Beta diversity was decreased in the microbiota of *S*+ (both intestinal sites) at D28 p.i. when compared to *S−*, although these changes were not statistically significant (Fig. 4). Nevertheless, at D50 p.i., microbial beta diversity in *S*+ mice (both intestinal sites) was significantly higher than that of *S−* mice at this time point (SI: *P* = 0.004, LI: *P = *0.008; Fig. 4).

### Expansion of populations of *Verrucomicrobiaceae* (*Akkermansia muciniphila*) and Lactobacillales and a reduction in Erysipelotrichia was associated with *S*. *mansoni* infection

**Figure 5:**
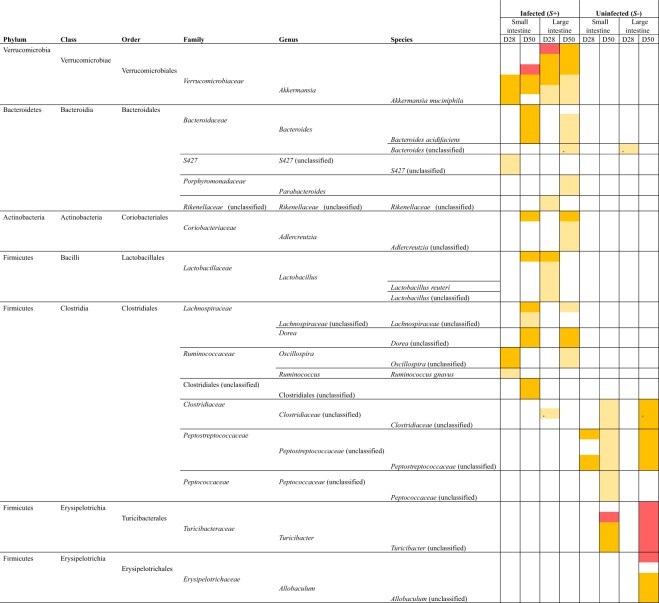
Bacterial taxa displaying significant differences in abundance between microbial profiles obtained from luminal content samples from mice experimentally infected with *Schistosoma mansoni* (*S*+) and uninfected controls (*S−*) based on linear discriminant analysis effect Size (LEfSe). For *S*+, datasets are separated for site (small and large intestine) and time point (28 and 50 days post-infection) and compared to the corresponding *S−* datasets. Colours correspond to Linear Discriminant Analysis (LDA) scores of 4.5 or higher (red), 4 to 4.5 (orange), and 3.5 to 4 (yellow).

Differences in the abundance of individual microbial taxa were detected between datasets from *S*+ and *S−* mice, at both intestinal sites and at both time-points (Fig. 5). In particular, a significant expansion in populations of bacteria of the Family *Verrucomicrobiaceae* (species *Akkermansia muciniphila*) was detected at both intestinal sites and at both time-points in *S*+ mice in comparison to *S−* (Fig. 5). Bacteria belonging to the *Lactobacillaceae* were also expanded in the LI of *S*+ mice at D28 p.i., while those belonging to the Orders Bacteroidales, Coriobacteriales and Clostridiales were expanded in either the SI i.e. *Bacteroides acidifaciens*, *Lachnospiraceae* and/or LI of *S*+ mice at D50 p.i., i.e. *Bacteroides acidifaciens*, *Parabacteroides*, *Adlercreutzia*, *Lachnospiraceae*, *Oscillospira* (Fig. 5). Conversely, a marked contraction of bacteria of the Class Erysipelotrichia was evident at both SI and LI of *S*+ mice at D50 p.i. compared to the uninfected counterparts (Fig. 5).

## Discussion

Increasing evidence links the immune-modulatory properties of helminth parasites to variations in the composition of the gut microbial communities of their mammalian hosts^12–15^. Attention has frequently focused on intestinal nematodes, likely because these helminths reside in contact with the microbiota of the alimentary tract^27,28^. While the adult stage of the schistosome does not reside within the lumen of the GI tract, infection with *S*. *mansoni* induces profound effects on gut immunity and homeostasis^26,29,30^; nevertheless, whether these effects are partly responsible for, or caused by, alterations in populations of gut microbes remains unanswered. In this study, we sought to characterise the fluctuations in the composition of the microbiome of the small and large intestine of a rodent model of schistosomiasis, before and after the occurrence of intestinal damage caused by transmission of the parasite eggs, in an effort to identify populations of bacteria with active roles in the complex host-parasite interplay. Taxonomic profiling of microbial communities using PCoA and CCA revealed strong associations between gut microbiota composition and stage of infection, thus providing further evidence of a (direct or immune-mediated) microbiota-modulatory role of parasitic helminths^12–25,31–39^. In particular, a significant decrease in microbial alpha diversity, which represents mean species diversity within a population of microbes^40^, was observed in both the SI and LI of *S*+ mice at D50 p.i. Accordingly, a recent study conducted in the Ivory Coast reported a lower level of alpha diversity in the faecal microbiota of *S*. *mansoni* infected children when compared to uninfected controls, albeit this difference did not reach statistical significance^41^. Decreases in gut microbial alpha diversity have been reported in association with GI helminth infections in several experimental systems, and to reflect a status of gut microbial dysbiosis following the onset of intestinal inflammation caused by infection^14,17,23,24^. In accordance with this hypothesis, the observation of a marked reduction in the gut microbial alpha diversity of *S*+ mice at D50 p.i. suggested a direct effect of inflammatory reactions resulting from migrating schistosome eggs. In contrast, microbial beta diversity was significantly increased in samples from *S*+ mice at D50 p.i. compared with *S−* mice, indicating that the gut microbiota of individual infected mice responded differently to the disruption of intestinal homeostasis caused by the helminth infection. A significant increase in microbial beta diversity had also been detected in the gut microbiota of laboratory rodents experimentally infected with the whipworm, *Trichuris suis* and the tapeworm *Hymenolepis diminuta*^17,34^, as well as in human volunteers from a Sri Lankan community infected with roundworms (i.e. hookworms, ascarids and/or whipworms)^31^. These reports, coupled with the findings from this study, suggest that increased beta diversity could represent a common feature characterising the gut microbiota of mammals parasitised by helminths, irrespective of parasite species and location in the GI tract. However, the status of infection, i.e. acute or chronic, as well as the overall parasite burden are likely to also affect helminth-induced changes in gut microbial diversity.

Significant differences in the relative abundance of a number of gut microbial taxa associated with helminth infection and intestinal inflammation were observed between *S*+ and *S−* mice, at both D28 p.i. and D50 p.i. In particular, at D28 p.i., bacterial populations from the Family *Lactobacillaceae* were significantly increased in the LI of *S*+ mice. The existence of direct and indirect relationships between lactobacilli and helminth parasites has been revealed based on data from experiments carried out in murine models of nematode infections^12,13,15–17,33,34^. In particular, in a key study, Reynolds *et al*.^13^ reported a marked increase in populations of *Lactobacillaceae* following infection of C57BL/6 mice with the intestinal nematode *Heligmosomoides polygyrus*. In turn, administration of *Lactobacillus* species to mice before helminth infection resulted in significantly increased worm burdens, which led the authors to hypothesise the occurrence of an immune-mediated, mutualistic relationship between selected bacteria and helminths^13^. Thus, higher levels of *Lactobacillaceae* at D28 p.i. in *S*+ mice compared to *S−* mice, could represent a microbiota-modulatory effect of *S*. *mansoni* which, in turn, may facilitate the establishment of chronic infection. Future studies in antibiotic-treated mice recolonised with a lactobacilli-deficient gut microbiota and subjected to infection with *S*. *mansoni* may assist research in this area. However, it is worth noting that no significant differences in the relative proportion of populations of *Lactobacillaceae* were detected in the gut microbiota of humans naturally or experimentally infected by GI nematodes when compared to uninfected subjects or others treated with anthelmintics^20–22,24,25,31^, and hence this mechanism might represent a rodent-specific effect of helminth infection on the host gut microbiota. Notably, the relative expansion of populations of *Lactobacillaceae* observed in the LI of *S*+ mice at D28 p.i. was no longer apparent at D50 p.i., likely suggesting that lactobacilli are negatively affected by the onset of inflammatory responses caused by the transiting schistosome eggs.

Besides lactobacilli, populations of *A*. *muciniphila* were significantly expanded in the microbiota from both the SI and LI of *S*+, and at both D28 p.i. and D50 p.i., when compared with the corresponding *S−* counterparts, with the largest difference recorded at the latter time point. *A*. *muciniphila* is a mucosal-dwelling anaerobic bacterium that degrades host mucins^42^; significantly expanded populations of *A*. *muciniphila* were also observed in the faecal microbiota of humans with mixed helminth infections^31^. The proliferation of *A*. *muciniphila* in the GI tract of infected mice may be directly linked to an increased production of mucins in response to schistosome infection; this hypothesis is supported by knowledge that transcription of *Muc5ac*, encoding for a gel-forming mucin, is up-regulated in tissues of schistosome-infected mice^43^. Indeed, mammalian mucins play a key role in the complex network of interactions occurring at the helminth-host interface^44–47^. For instance, mice infected with *Echinostoma trivolvis* displayed a dramatically increased production of host mucins, which was crucial to the expulsion of the parasites^48^. In addition to an association with increased host mucin production, *A*. *muciniphila* adheres to the intestinal epithelium and strengthens enterocyte monolayer integrity *in vitro*^49^. Thus, it is plausible that increased levels of *A*. *muciniphila* may play a potential protective role against the disruption of gut epithelial barrier function caused by granuloma formation.

At D50 p.i., in concomitance with schistosome egg migration and granuloma formation, bacterial taxa that have been previously associated with intestinal inflammation, e.g. of the Family *Lachnospiraceae* (i.e. *Dorea*) and genus *Bacteroides* (i.e. *Bacteroides acidifaciens*) were significantly more abundant in the gut microbiota of *S*+ compared to *S−* mice. Expanded populations of *Dorea* have been reported in irritable bowel syndrome patients^50^, as well during intestinal inflammation^51^, whilst *Bacteroides acidifaciens* was enriched in a mouse model of colitis^52^. Together with the increased levels of beta- and decreased levels of alpha diversity detected in *S*+ mice at D50 p.i., these findings lend credit to the hypothesis that a significant disruption of gut microbial homeostasis occurred at this time-point, and that this disruption contributed to the intestinal pathogenesis during *S*. *mansoni* infection. This hypothesis is supported by findings from Holzscheiter and colleagues^26^, who reported a significant reduction of intestinal inflammation and intestinal granuloma development in *S*. *mansoni* infected mice after antibiotic treatment.

In contrast to the above-mentioned bacteria, those belonging to the Class Erysipelotrichia (Orders Turicibacterales and Erysipelotrichales) were significantly reduced in *S*+ mice at D50 p.i. when compared with uninfected mice. To our knowledge, information is not available on the relationships between this group of bacteria and infections by GI helminths or schistosomes; however, in immune-deficient mice, a clear link between Turicibacterales and host immune dysfunction has been described^53,54^. For example, species of Turicibacterales are abundant in the gut microbiota of wild type mice, but completely absent from the gut of mice with defective immune responses (CD45−/− knockout) and mice lacking an adaptive immune system (RAG−/− knockout)^54^. Accordingly, we suggest that the lower abundance of Turicibacterales observed in *S*+ compared to *S−* mice could be due to disturbances of mucosal immune functions during *S*. *mansoni* infection. However, it remains unclear whether and/or how a contraction in populations of Erysipelotrichia might have an impact on the outcome of *S*. *mansoni* infection.

In conclusion, our study shows that infection of mice with *S*. *mansoni* infection is associated with profound comprehensive shifts in the global composition of the host gut microbiota, and that the changes are indicative of dysbiosis accompanying egg migration across the intestinal wall and granuloma formation. Many of the specific infection-related alterations to the microbiota involved bacterial taxa which are linked to host immune-regulation or inflammation, suggesting that the balance between immune-regulatory and pro-inflammatory bacterial taxa during schistosomiasis play a key role in determining the effective establishment of the infection, and/or severity of the disease resulting from host immune responses to infection.

## Materials and Methods

### Ethics statement

Swiss-Webster female mice were obtained from the NIAID Schistosomiasis Resource Center, Rockville, MD, via distribution through BEI Resources, and housed at the Animal Research Facility of the George Washington University. The latter is accredited by the American Association for Accreditation of Laboratory Animal Care (AAALAC no. 000347) and holds an Animal Welfare Assurance by the National Institutes of Health, Office of Laboratory Animal Welfare (OLAW: A3205-01). Procedures described here were performed in accordance with the Guide for the Care and Use of Laboratory Animals, and approved by the Institutional Animal Care and Use Committee of the George Washington University, protocol number A137.

### Infection of mice, sample collection and parasitological analyses

Ten Swiss-Webster female mice (*S*+) were percutaneously infected with 200 *S*. *mansoni* (NMRI strain) cercariae by tail exposure, as described^55^, while 10 age- and gender-matched mice remained uninfected and were included as controls (*S−*). For each *S*+ and *S−*, five mice were euthanised 28 days after infection (D28 p.i., before the start of egg laying by sexually mature *S*. *mansoni* females) by an overdose of Euthasol (sodium pentobarbital and sodium phenytoin, ~40 mg per mouse) (Virbac Corporation, Fort Worth, TX) delivered by intraperitoneal injection. The remaining mice (n = 5 for each *S*+ and *S−*) were euthanised, as above, at day 50 p.i. (D50 p.i., corresponding with ongoing egg laying and extensive granuloma formation). Adult *S*. *mansoni* were recovered from each mouse in *S*+ by portal perfusion, and livers were removed for egg isolation and counting as described previously^56^. Luminal content samples were collected from sections of the small (SI) and large intestines (LI) of individual *S*+ and *S−* mice at each D28 p.i. and D50 p.i. under sterile conditions. Briefly, after opening the abdominal cavities, the intestines were incised longitudinally with a sterile razor blade and SI and LI luminal contents were transferred directly into sterile tubes. These were snap frozen on dry ice and stored at −80 °C until further use. The experiment was repeated twice for data validation, resulting in a total of 20 *S*+ and 20 *S−* mice included in the study.

### DNA extraction and bacterial 16S rRNA gene sequencing

Genomic DNA was extracted directly from each luminal content sample, as well as two no-template negative control samples, using the PowerSoil DNA Isolation Kit (MO BIO Laboratories, Carlsbad, CA, USA), according to manufacturers’ instructions, within one month from sample collection. High-throughput sequencing of the V4 hypervariable region of the prokaryotic 16S rRNA gene was performed on an Illumina MiSeq platform. The V4 region was PCR-amplified using universal primers^57^, that contained the Illumina adapter overhang nucleotide sequences, using the NEBNext hot start high-fidelity DNA polymerase (New England Biolabs), 2 ng/μl of template DNA and the following thermocycling protocol: 2 min at 98 °C, 20 cycles of 15 s at 98 °C – 30 s at 63 °C – 30 s at 72 °C, and a final elongation step of 5 min at 72 °C. Amplicons were purified using AMPure XP beads (Beckman Coulter) and the NEBNext hot start high-fidelity DNA polymerase was used for the index PCR with Nextera XT index primers (Illumina), with thermocycling as follows: 3 min at 95 °C, 8 cycles of 30 s at 95 °C – 30 s at 55 °C – 30 s at 72 °C, and 5 min at 72 °C. The indexed samples were purified using AMPure XP beads, quantified using the Qubit dsDNA high sensitivity kit (Life Technologies), and equal quantities from each sample pooled. The pooled library was quantified using the NEBNext library quantification kit (New England Biolabs) and sequenced using the v3 chemistry (301 bp paired-end reads). The raw sequences are available from the Mendeley database at 10.17632/y8c7vpc8zp.1.

### Bioinformatics and statistical analyses

Paired-end Illumina reads were trimmed for 16S rRNA gene primer sequences using Cutadapt (https://cutadapt.readthedocs.org/en/stable/) and sequence data were processed using the Quantitative Insights Into Microbial Ecology (QIIME-1.9.1) software suite^58^. Successfully merged reads were quality filtered in QIIME using default settings. Thereafter, sequences were clustered into OTUs on the basis of similarity to annotated bacterial sequences available in the Greengenes database (v13.8; http://greengenes.secondgenome.com/; 97% sequence similarity cut-off) using the UCLUST software within QIIME. Sequences that could not be matched to references in the Greengenes database were clustered *de novo* based on pair-wise sequence identity (97% sequence similarity cut-off). The first selected cluster seed was considered as the representative sequence of each OTU. Taxonomy assignment of representative sequences was accomplished with the UCLUST software. Singleton OTUs were removed prior to downstream analyses. Cumulative-sum scaling (CSS) was applied, followed by log2 transformation to account for the non-normal distribution of taxonomic counts data. Statistical analyses were executed using the Calypso software^59^ (cgenome.net/calypso/). Samples were ordinated using Principle Coordinates Analysses (PCoA) based on weighted UniFrac distances. CCA was then performed, including infection status and time point as explanatory variables. In addition, Permutational Multivariate Analyses of Variance Using Distance Matrices (Adonis)^60^ was used to calculate the portion of variability in the data explained by the explanatory variables, and a biplot was generated in R^61^. Following rarefaction of raw data, differences in microbial alpha diversity (Shannon diversity) and richness between *S*+ and *S−* groups, as well as in the abundance of individual taxa, were evaluated using ANOVA. Beta diversity was calculated using weighted UniFrac distances and differences in beta diversity were calculated using Analysis of Similarity (ANOSIM)^62^. Differences in the composition of the microbiota between groups were assessed using the Linear discriminant analysis Effect Size (LEfSe) workflow^63^. Following the completion of bioinformatics analyses using QIIME (QIIME-1.9.1), QIIME2 was released (QIIME2-2018.4; https://qiime2.org). Therefore, in order to ensure that no substantial differences in findings could be detected when performing data analysis and annotation using this updated software, we undertook separate bioinformatic analyses and compared the resulting findings with those originally obtained using QIIME-1.9.1. In addition, reproducibility of findings was also ensured by replacing the Greengenes database with the SILVA reference database (https://www.arb-silva.de/download/archive/qiime; Silva_132) for sequence data annotation. Briefly, no major differences were detected between findings generated with QIIME-1.9.1 or QIIME2-2018.4 and using the Greengenes or SILVA repository as reference databases. Thus, the results from these additional analyses are not shown in the main article. Nevertheless, individual data files displaying findings from these analyses (e.g. differences in microbial alpha and beta diversity between mouse groups and infection time points, as well as differences in the relative abundances of individual OTUs) are available from the Mendeley database at 10.17632/y8c7vpc8zp.1.

## Electronic supplementary material

Supplementary Material

## References

[1] Hotez PJ (2008). Helminth infections: The great neglected tropical diseases. J. Clin. Invest..

[2] Garba Djirmay A, Montresor A (2016). Schistosomiasis and soil-transmitted helminthiases: number of people treated in 2015. WHO..

[3] Colley DG, Bustinduy AL, Secor WE, King CH (2014). Human schistosomiasis. Lancet..

[4] Van der Werf MJ (2003). Quantification of clinical morbidity associated with schistosome infection in sub-Saharan Africa. Acta Trop..

[5] Ross AG (2002). Schistosomiasis. N. Engl. J. Med..

[6] Gryseels B, Polman K, Clerinx J, Kestens L (2006). Human schistosomiasis. Lancet..

[7] Pearce EJ, MacDonald AS (2002). The immunobiology of schistosomiasis. Nat. Rev. Immunol..

[8] Wilson MS (2007). Immunopathology of schistosomiasis. Immunol. Cell. Biol..

[9] Turner JD, Narang P, Coles MC, Mountford AP (2012). Blood flukes exploit Peyer’s Patch lymphoid tissue to facilitate transmission from the mammalian host. PLoS Pathog..

[10] Warren KS (1978). The pathology, pathobiology and pathogenesis of schistosomiasis. Nature..

[11] Haeberlein S (2017). Schistosome egg antigens, including the glycoprotein IPSE/alpha-1, trigger the development of regulatory B cells. PLoS Pathog..

[12] Fricke WF (2015). Type 2 immunity-dependent reduction of segmented filamentous bacteria in mice infected with the helminthic parasite *Nippostrongylus brasiliensis*. Microbiome..

[13] Reynolds LA (2014). Commensal-pathogen interactions in the intestinal tract: *Lactobacilli* promote infection with, and are promoted by, helminth parasites. Gut Microbes..

[14] Cattadori IM (2016). Impact of helminth infections and nutritional constraints on the small intestine microbiota. PLoS One..

[15] Rausch S (2013). Small intestinal nematode infection of mice is associated with increased enterobacterial loads alongside the intestinal tract. PLoS One..

[16] Walk ST, Blum AM, Ewing SA, Weinstock JV, Young VB (2010). Alteration of the murine gut microbiota during infection with the parasitic helminth *Heligmosomoides polygyrus*. Inflamm. Bowel. Dis..

[17] Holm JB (2015). Chronic *Trichuris muris* infection decreases diversity of the intestinal microbiota and concomitantly increases the abundance of *Lactobacilli*. PLoS One..

[18] McKenney EA (2015). Alteration of the rat cecal microbiome during colonization with the helminth *Hymenolepis diminuta*. Gut Microbes..

[19] Plieskatt JL (2013). Infection with the carcinogenic liver fluke *Opisthorchis viverrini* modifies intestinal and biliary microbiome. FASEB J..

[20] Giacomin P (2015). Experimental hookworm infection and escalating gluten challenges are associated with increased microbial richness in celiac subjects. Sci. Rep..

[21] Giacomin P (2016). Changes in duodenal tissue-associated microbiota following hookworm infection and consecutive gluten challenges in humans with coeliac disease. Sci. Rep..

[22] Lee, S. C. *et al*. Helminth colonization is associated with increased diversity of the gut microbiota. *PLoS Negl.**Trop. Dis.* **8**, e2880, 10.1371/journal.pntd.0002880 (2014). 10.1371/journal.pntd.0002880 PMC4031128 24851867

[23] Houlden A (2015). Chronic *Trichuris muris* infection in C57BL/6 mice causes significant changes in host microbiota and metabolome: Effects reversed by pathogen clearance. PLoS One..

[24] Cooper, P. *et al*. Patent human infections with the whipworm, *Trichuris trichiura*, are not associated with alterations in the faecal microbiota. *PLoS One*. **8**, 10.1371/journal.pone.0076573 (2013). 10.1371/journal.pone.0076573 PMC3790696 24124574

[25] Cantacessi C (2014). Impact of experimental hookworm infection on the human gut microbiota. J. Infect. Dis..

[26] Holzscheiter M (2014). Lack of host gut microbiota alters immune responses and intestinal granuloma formation during schistosomiasis. Clin. Exp. Immunol..

[27] Peachey LE, Jenkins TP, Cantacessi C (2017). This gut ain’t big enough for both of us. Or is it? Helminth-microbiota interactions in veterinary species. Trends Parasitol..

[28] Zaiss MM, Harris NL (2016). Interactions between the intestinal microbiome and helminth parasites. Parasite Immunol..

[29] Mayer JU (2017). Different populations of CD11b(+) dendritic cells drive Th2 responses in the small intestine and colon. Nat. Commun..

[30] Pearce EJ (2004). Th2 response polarization during infection with the helminth parasite *Schistosoma mansoni*. Immunol. Rev..

[31] Jenkins TP (2017). Infections by human gastrointestinal helminths are associated with changes in faecal microbiota diversity and composition. PLoS One.

[32] Broadhurst MJ (2012). Therapeutic helminth infection of macaques with idiopathic chronic diarrhea alters the inflammatory signature and mucosal microbiota of the colon. PLoS Pathog..

[33] Su C (2017). Helminth-induced alterations of the gut microbiota exacerbate bacterial colitis. *Mucosal Immunol*. 10.1038/mi..

[34] Kreisinger, J., Bastien, G., Hauffe, H. C., Marchesi, J. & Perkins, S. E. Interactions between multiple helminths and the gut microbiota in wild rodents. *Philos*. *Trans*. *R*. *Soc*. *Lond*. *B Biol*. *Sci*. **370** (2015). 10.1098/rstb.2014.0295 PMC4528493 26150661

[35] Li RW (2012). Alterations in the porcine colon microbiota induced by the gastrointestinal nematode *Trichuris suis*. Infec.t Immun..

[36] Li RW (2016). The effect of helminth infection on the microbial composition and structure of the caprine abomasal microbiome. Sci. Rep..

[37] Duarte AM (2016). Helminth infections and gut microbiota - a feline perspective. Parasit. Vectors..

[38] Slapeta J, Dowd SE, Alanazi AD, Westman ME, Brown GK (2015). Differences in the faecal microbiome of non-diarrhoeic clinically healthy dogs and cats associated with *Giardia duodenalis* infection: Impact of hookworms and coccidia. Int. J. Parasitol..

[39] Li RW, Wu S, Li W, Huang Y, Gasbarre LC (2011). Metagenome plasticity of the bovine abomasal microbiota in immune animals in response to *Ostertagia ostertagi* infection. PLoS One.

[40] Tuomisto H (2010). A diversity of beta diversities: Straightening up a concept gone awry: Defining beta diversity as a function of alpha and gamma diversity. Ecography..

[41] Schneeberger PHH (2018). Investigations on the interplays between *Schistosoma mansoni*, praziquantel and the gut microbiome. Parasit. Vectors..

[42] Derrien M, Collado MC, Ben-Amor K, Salminen S, de Vos WM (2008). The Mucin degrader *Akkermansia muciniphila* is an abundant resident of the human intestinal tract. Appl. Environ. Microbiol..

[43] Scheer S (2014). *S*. *mansoni* bolsters anti-viral immunity in the murine respiratory tract. PLoS One.

[44] Theodoropoulos G, Hicks SJ, Corfield AP, Miller BG, Carrington SD (2001). The role of mucins in host-parasite interactions: Part II - helminth parasites. Trends Parasitol..

[45] Cortes A (2015). *Echinostoma caproni* (Trematoda): Differential *in vivo* mucin expression and glycosylation in high- and low-compatible hosts. Parasite Immunol..

[46] Munoz-Antoli C (2016). Interleukin-25 induces resistance against intestinal trematodes. Sci. Rep..

[47] Cancela M (2015). Fasciola hepatica mucin-encoding gene: expression, variability and its potential relevance in host-parasite relationship. Parasitology..

[48] Fujino T, Ichikawa H, Fukuda K, Fried B (1998). The expulsion of *Echinostoma trivolvis* caused by goblet cell hyperplasia in severe combined immunodeficient (SCID) mice. Parasite..

[49] Reunanen J (2015). *Akkermansia muciniphila* adheres to enterocytes and strengthens the integrity of the epithelial cell layer. Appl. Environ. Microbiol..

[50] Rajilić–Stojanović, M. *et al*. Global and deep molecular analysis of microbiota signatures in fecal samples from patients with irritable bowel syndrome. *Gastroenterology*. **141**, 1792–1801, 10.1053/j.gastro.2011.07.043. 10.1053/j.gastro.2011.07.043 21820992

[51] Jenq RR (2012). Regulation of intestinal inflammation by microbiota following allogeneic bone marrow transplantation. J. Exp. Med..

[52] Bloom SM (2011). Commensal *Bacteroides* species induce colitis in host-genotype-specific fashion in a mouse model of inflammatory bowel disease. Cell Host Microbe..

[53] Presley LL, Wei B, Braun J, Borneman J (2010). Bacteria associated with immunoregulatory cells in mice. Appl. Environ. Microbiol..

[54] Dimitriu PA (2013). Temporal stability of the mouse gut microbiota in relation to innate and adaptive immunity. Environ. Microbiol. Rep..

[55] Tucker MS, Karunaratne LB, Lewis FA, Freitas TC, Liang YS (2013). Schistosomiasis. Curr. Protoc. Immunol..

[56] Mann VH, Morales ME, Rinaldi G, Brindley PJ (2010). Culture for genetic manipulation of developmental stages of *Schistosoma mansoni*. Parasitology..

[57] Klindworth, A. *et al*. Evaluation of general 16S ribosomal RNA gene PCR primers for classical and next-generation sequencing-based diversity studies. *Nucleic Acids Res*. **41**, e1, 10.1093/nar/gks808 (2013). 10.1093/nar/gks808 PMC3592464 22933715

[58] Caporaso, J. G. *et al*. QIIME allows analysis of high-throughput community sequencing data. *Nature Meth*. **7**, 10.1038/nmeth.f.303 (2010). 10.1038/nmeth.f.303 PMC3156573 20383131

[59] Zakrzewski M (2017). Calypso: a user-friendly web-server for mining and visualizing microbiome-environment interactions. Bioinformatics..

[60] Anderson, M. J. A new method for non‐parametric multivariate analysis of variance. *Austral Ecology*. **26**, 32–46 10.1111/j.1442-9993.2001.01070.pp.x.

[61] Oksanen, J. *et al*. Vegan: Community Ecology Package. R package version 2.3-3, http://CRAN.R-project.org/package=vegan (2016).

[62] Clarke KR (1993). Non-parametric multivariate analyses of changes in community structure. Austral. Ecology..

[63] Segata, N. *et al*. Metagenomic biomarker discovery and explanation. *Genome Biol*. **12**, 10.1186/gb-2011-12-6-r60 (2011). 10.1186/gb-2011-12-6-r60 PMC3218848 21702898

