# Supplementary material for: Schistosoma mansoni infection is associated with quantitative and qualitative modifications of the mammalian intestinal microbiota
Source: Sci Rep. 2018 Aug 13;8:12072. doi: 10.1038/s41598-018-30412-x (PMC6089957; doi:10.1038/s41598-018-30412-x)
Supplement: Supplementary file 1 — Supplementary Material [file 41598_2018_30412_MOESM1_ESM.pdf]

Supplementary information

Schistosoma mansoni infection is associated with quantitative and qualitative modifications of the mammalian intestinal microbiota

Timothy P. Jenkins[1†], Laura E. Peachey[1†], Nadim J. Ajami[2], Andrew S. MacDonald[3], Michael H. Hsieh[4,5,6], Paul J. Brindley[7*], Cinzia Cantacessi[1*], Gabriel Rinaldi[7,8*]

[1] Department of Veterinary Medicine, University of Cambridge, Cambridge CB3 0ES, UK; [2] Alkek Center for Metagenomics and Microbiome Research, Department of Molecular Virology and Microbiology, Baylor College of Medicine, Houston, Texas, USA; [3] Manchester Collaborative Centre for Inflammation Research, Division of Infection, Immunity and Respiratory Medicine, School of Biological Sciences, Faculty of Biology, Medicine and Health, University of Manchester, Manchester Academic Health Science Centre, Manchester M13 9PL, UK; [4] Biomedical Research Institute, Rockville, Maryland, USA; [5] Department of Urology, School of Medicine and Health Sciences, George Washington University, Washington, USA; [6] Children's National Health System, Washington, District of Columbia, USA; [7] Department of Microbiology, Immunology & Tropical Medicine, and Research Center for Neglected Diseases of Poverty, School of Medicine & Health Sciences, George Washington University, Washington DC, 20037, USA; [8] Wellcome Sanger Institute, Wellcome Genome Campus, Hinxton, CB10 1SA, UK

[†,*]Equal contributions.

Supplementary Figure S1.

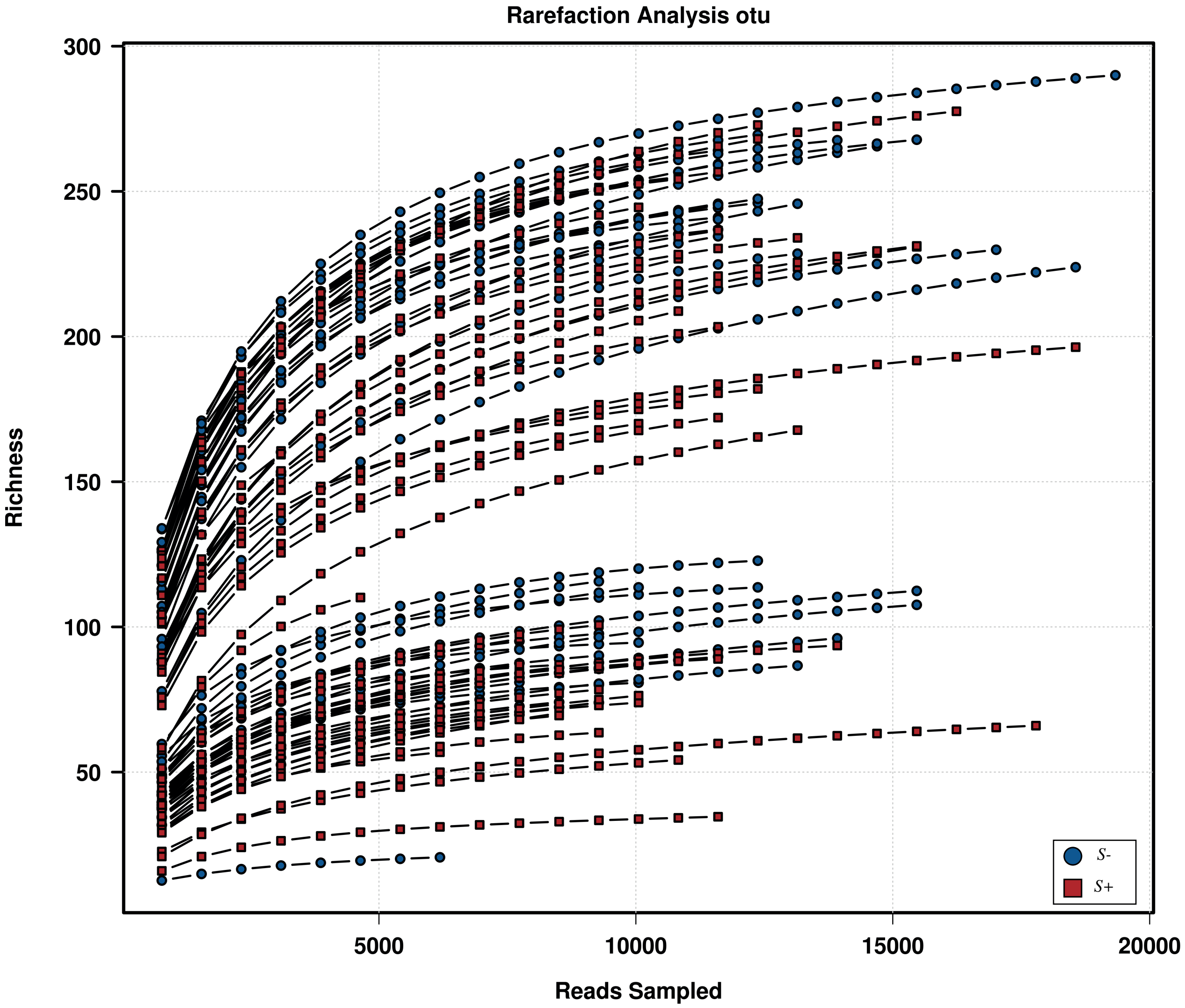

Supplementary Fig. S1 | Rarefaction curves for gut microbial communities from luminal contents of mice infected with *Schistosoma mansoni* (*S*+) and that of uninfected control mice (*S*-).

Supplementary Figure S2.

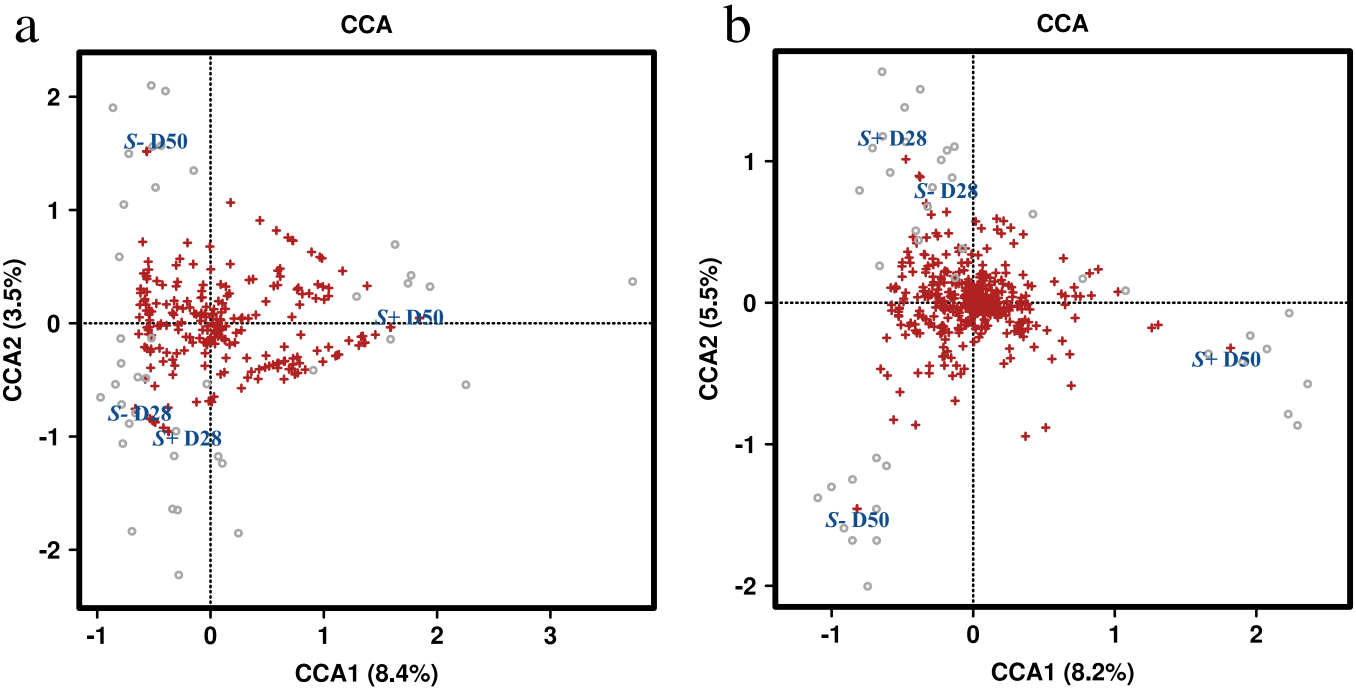

Supplementary Fig. S2 | Canonical Correspondence Analyses (CCA) Biplot, demonstrating the portion of variability in the data attributable to the explanatory variables. The gut microbial profiles of luminal content samples from the small and large intestine of mice infected by *Schistosoma mansoni* (*S*+) at 28 and 50 days post-infection (D28 and D50, respectively), as well as of uninfected controls (*S*-) ordinated by Canonical Correspondence Analysis (CCA) (a: small intestine; b: large intestine).
